# Identification of MATN3 as a Novel Prognostic Biomarker for Gastric Cancer through Comprehensive TCGA and GEO Data Mining

**DOI:** 10.1155/2021/1769635

**Published:** 2021-12-02

**Authors:** Pan Wang, Wei-sheng Xiao, Yue-hua Li, Xiao-ping Wu, Hong-bo Zhu, Ye-ru Tan

**Affiliations:** ^1^Department of Medical Oncology, The First Affiliated Hospital of Hengyang Medical School of University of South China, Hengyang, Hunan, China; ^2^Department of Gastroenterology, The First Affiliated Hospital of Hengyang Medical School of University of South China, Hengyang, Hunan, China

## Abstract

Gastric cancer (GC) is still a vital malignant cancer across the world with unsatisfactory prognostic results. Matrilin-3 (MATN3) is a member of the extracellular matrix (ECM) protein family. The present research intends to explore the expression level of MATN3 in patients with GC and to explore the prognosis significance of MATN3. In this study, we observed that the MATN3 expression was remarkably upregulated in GC samples in contrast to noncancer samples. Clinical analyses unveiled that high MATN3 expression was related to age, tumor status, and clinical stages. Survival analyses unveiled that patients with high MATN3 expression displayed a poorer overall survival and progression-free survival than those with low MATN3 expression. The AUC of the relevant ROC curve for 1 year, 3 years, and 5 years of survival is 0.571, 0.596, and 0.720, separately. Multivariate assays revealed that MATN3 expression and stage were independent predictors of poor prognosis of GC patients. A meta-analysis unveiled that high MATN3 expression was tightly associated with better overall survival. Overall, our data indicated that MATN3 may have a diagnostic and prognostic value for patients with advanced gastric cancer and assist to improve clinical outcomes for GC patients.

## 1. Introduction

Gastric cancer (GC) is the second most commonly seen death reason from tumor worldwide, accounting for more than 720 thousand mortalities each year [1]. Despite the fact that there have been certain progresses in diagnosis and treatment of early-stage GC sufferers, it is quite difficult to cure advanced GC [2, 3]. The clinical stage, on the foundation of the TNM categorization system, at the time of diagnosis is at present the most vital prognosis factor, and the molecule-level causal link participating in the development and metastatic activities of GC is still elusive [4, 5]. For that reason, the determination of tumor genesis-related molecule-level markers (with high sensitivity and specificity) which could significantly determine the clinic features of GC and precisely forecast the relapse and prognostic results is a vital objective of GC research.

MATN3 (Matrilin-3), also named DIPOA, OADIP, or EDM5, is a protein coding gene, which encodes a component of von Willebrand factor A domain with protein family [6, 7]. This protein family is considered to participate in the forming of filamentous nets in the ECMs of a variety of tissues [8, 9]. Past researches have displayed that MATN3 was discovered in matrices created by cultivated chondrosarcoma cells and participated in the developmental process of cartilages and bones [10, 11]. MATN3 mutations are related to commonly seen bone illnesses and scarce dyschondroplasia [12, 13]. In addition, several studies have reported the distinct dysregulation of MATN3 in several tumors, such as pancreatic ductal adenocarcinoma and osteosarcoma [14, 15]. However, the expression and function of MATN3 in GC remained largely unclear.

The present research intended to evaluate the diagnosis and prognosis significance of MATN3 expressing in mankind GC on the foundation of the TCGA data. Our team completed a comprehensive meta-analysis to evaluate the general prognosis significance of MATN3 via the data from 2 publicly available databases. Eventually, our team explored the biology process of MATN3, where MATN3 is involved via genetic enrichment assay, to investigate the relevant causal link of MATN3 in GC.

## 2. Materials and Methods

### 2.1. TCGA and Microarray Genetic Profiling Data Assay

The TCGA GC samples (*n* = 375) and nontumor specimens (*n* = 32) RNA sequence data were acquired from TCGA datasets (https://portal.gdc.cancer.gov/repository). Another GC RNA sequence data (GSE84437) including 433 GC patients were acquired from GEO [16]. These data were subjected to pre-processing via R software. The clinical data of the GC patients were also obtained from the TCGA and GEO databases. Since the data were provided by the TCGA and GEO databases, the approval from the Ethics Committee was not required.

### 2.2. Meta-Analysis

The PubMed, Web of Science, and Embase databases were retrieved in an all-round way to acquire the entire published researches on the relationship between MATN3 and the prognostic results of GC. Given that the present report marks the first research to explore the prognosis effect of MATN3 for GC, no past researches were acquired from these databases. For that reason, our team was able to use the gathering analysis to evaluate the general prognosis value of MATN3 in GC sufferers from two datasets. Integrated HR and 95% CI were computed to assess the relationship of MATN3 expression with the prognostic results of GC sufferers. The inhomogeneity of 4 datasets was evaluated by the *Q* test (*I*^2^ statistics). A fixed-effect model will be chosen for the combination if no evident inhomogeneity (*I*^2^ < 50%), whereas a stochastic effect model will be employed. The gathering analysis was performed via R program.

### 2.3. Functional Assay

For the purpose of verifying the potential roles of underlying targets, the data were assayed via function enrichment. GO is a broadly utilized tool to annotate genes with functions, particularly for MF, BP, and CC [17]. KEGG enrichment assay is a useful resource for analysis-based research of genetic roles and related high-level genomic function [18]. For the purpose of understanding the function of MATN3 in GC progression, ClusterProfiler package 3.12.0 in R was utilized with the aim of analyzing the GO function of underlying targets and enriching the KEGG pathway [19]. “*p* < 0.05” was adopted as the selection standard.

### 2.4. Statistical Analysis

The entire statistics were finished via the R program 3.6.2. The diversity between diverse groups was evaluated via the Mann–Whitney *U* test and classification data were analyzed via the chi-square test. Survival curves were drawn by the K-M approach and evaluated via log-rank test. The time-reliant ROC curve was employed to identify the prognosis effect via contrasting the AUC through R package “pROC” [20]. In addition, 10-fold cross-approach was employed for ROC verification and AUC data computation. Univariable and multivariable assays were finished by the Cox proportion risk regressive assay. The outcomes with *p* < 0.05 had significance on statistics, offering dependability for the assay.

## 3. Results

### 3.1. The Upregulation of MATN3 in GC and Its Clinical Significance

**Figure 1:**
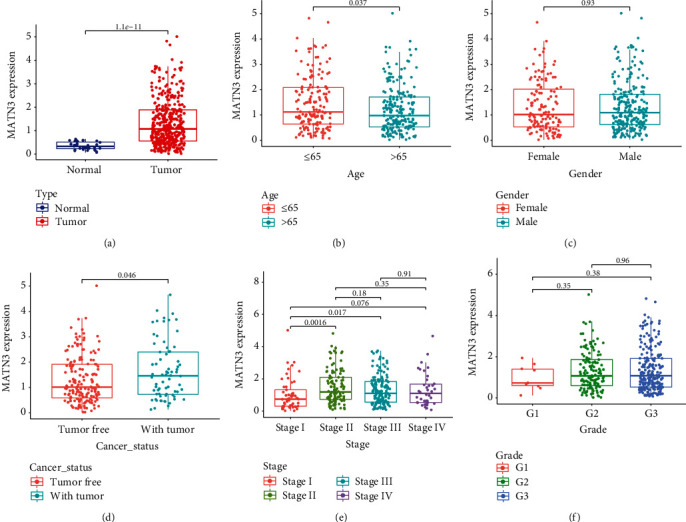
The distinct upregulation of MATN3 and its association with clinical features. (a) MATN3 expression was determined in GC specimens and non-tumor specimens using TCGA datasets. MATN3 mRNA levels were classified by age (b), sex (c), cancer status(d), stage(e), and grade(f).

For the purpose of exploring the biology-wise significance of MATN3 in GC patients, our team analyzed the TCGA datasets and found that the expression of MATN3 was remarkably elevated in GC samples in contrast to noncancer samples (Figure 1(a)). Clinical assays revealed that the dysregulation of MATN3 expression was associated with age of GC patients (Figure 1(b)), while it did not associate with gender of GC patients (Figure 1(c)). Importantly, patients with positive tumor status and advanced clinical stages exhibited a higher level of MATN3 (Figures 1(d) and 1(e)). However, we did not observe a distinct association between the MATN3 expression and grade (Figure 1(f)).

### 3.2. The Prognostic Performance of MATN3 Expression in GC Patients

**Figure 2:**
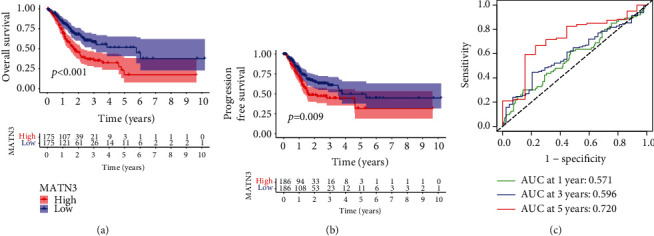
Survival assays of GC patients from TCGA datasets based on MATN3 mRNA expression (high versus low). (a) K-M curves for OS of 350 GC sufferers. (b) K-M curves for survival without development of 372 GC sufferers. (c) Survival-reliant ROC curves confirm the prognosis value of MATN3-based prognosis indexes.

**Figure 3:**
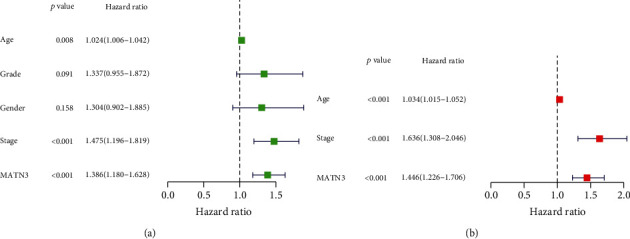
The prognosis value of MATN3 expression in GC patients. (a, b) Forest plot exhibited the HR with 95% CI of MATN3 in GC based on the univariable (a) and multivariable (b) assays.

For the purpose of exploring the prognosis performance of MATN3 expression in GC sufferers, our team separated the entire GC sufferers into 2 groups (high and low) as per the mean expression of MATN3. Survival assays unveiled that the sufferers with high MATN3 levels displayed an inferior overall survival (*p* < 0.001, Figure 2(a)) and progression-free survival (*p* = 0.009, Figure 2(b)) in contrast to the low MATN3 levels. The AUC of the relevant ROC curve for 1 year, 3 years, and 5 years of survival is 0.571, 0.596, and 0.720, separately, which revealed that the prognosis indicator on the foundation of MATN3 expression had some possibilities to predict survival (Figure 2(c)). More importantly, in multivariate OS analysis, we found that MATN3 expression (HR = 1.446; 95% CI, 1.226-1.706; *p* < 0.004), stage (HR = 1.636, 95% CI, 1.308-2.046; *p* < 0.001), and age (HR = 1.034; 95% CI, 1.015-1.052; *p* < 0.004) were independent predictors of poor prognosis (Figures 3(a) and 3(b)).

### 3.3. Meta-Analysis and Predictive Performance of MATN3 Expression

**Figure 4:**
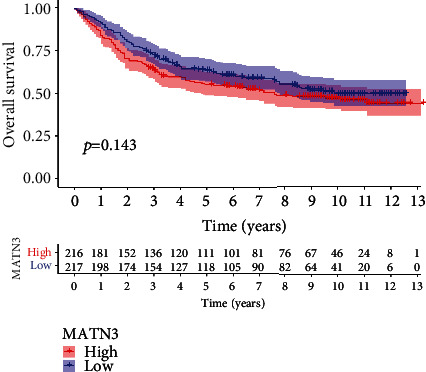
K-M curves of the OS of 433 GC sufferers from GSE84437.

**Figure 5:**
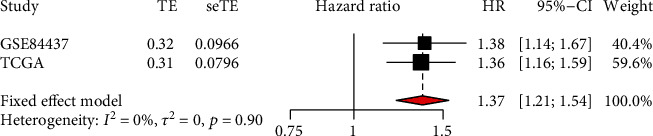
Forest plot of high MATN3 expressions with improved overall survival sufferers from two datasets.

Then, we analyzed the survival significance of MATN3 expression using GSE84437. Although no distinct association was observed in GC patients with dysregulated MATN3 expression, sufferers with high MATN3 expressions exhibited an inferior OS in clinical trend (Figure 4). Then, we performed meta-analysis using 783 patients from the TGCA datasets and GSE84437. As shown in Figure 5, the pooled HR as well as 95% CI for the relationship between high MATN3 expressions and overall survival in 783 GC sufferers was 1.37 (1.21-1.54), and there was not remarkable inhomogeneity between the two datasets. For that reason, our team came to the conclusion with confidence that high MATN3 expressions was a potent predicting factor of better overall survival amongst GC sufferers.

### 3.4. MATN3-Related Signaling Pathways in GC

**Figure 6:**
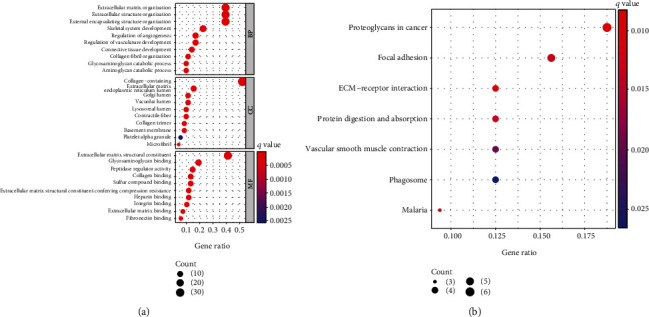
(a) GO is assayed and presents the Top 10 of BP, CC, and MF. (b) KEGG pathways are assayed, and the top 7 pathways are mapped.

For the purpose of exploring the function of MATN3 in GC, our team screened dysregulated genes in GC specimens in the high MATN3 expression group. Then, we performed GO and KEGG assays using these genes. Top 30 of GO enriching were listed in Figure 6(a). GO enriching reveals that the biological processes of differential genes primarily participate in ECM organization, exocellular framework organization, extracellular matrix, and extracellular matrix structural constituent. KEGG enriching displays that pathways of differential genes primarily include pathways in proteoglycans in tumor, focal adhesion, protein digestion and absorption, vascular smooth muscle contraction, phagosome, and malaria (Figure 6(b)).

## 4. Discussion

Recently, the development in genomics, proteomics, and metabolomics techniques has discovered critical molecule-level activities in the process of GC carcinogenesis, which has triggered the identification of new GC markers, such as gene and epigene variations, mRNA, noncoding RNA, posttranslation protein modifications, and metabolins [21–23]. Those diverse markers might be detected in blood, sera, plasma, urine, cancer tissues, or neighboring benign hepatic tissues [24, 25]. mRNAs are deemed as promising markers as they are detectable, steady, and tightly related to clinic results [26, 27].

MATN3, as a component of the matrilin protein family, is a noncollagenous ECM [9]. It has attracted extensive attention in recent years, especially in bone- and cartilage-associated areas [10, 28]. The mutation of MATN3 gene may lead to some diseases such as multiple epiphyseal dysplasia (MED), epiphyseal dysplasia (BHMED), and epiphyseal dysplasia of the vertebral body (SEMD) [6, 29, 30]. Up till now, despite the fact that substantial research on MATN3 has been focused on epiphysis illness, little research has been completed with regard to more situations like malignancies. In the present research, our team observed that MATN3 expressing was distinctly upregulated in GC based on the results of the TCGA datasets. Higher levels of MATN3 were associated with advanced tumor stages and positive cancer status. Survival assays confirmed that high MATN3 expressions was related to inferior prognostic results of GC sufferers. The present gathering analysis involving 783 GC sufferers from two databases revealed that high MATN3 expressions acted as an independent prognosis variate of overall survival in GC sufferers. Moreover, KEGG enrichment analysis showed that proteoglycans, focal adhesion, and ECM-receptor interaction were the most significant pathways. Our findings highlighted the potential of MATN3 used as a novel biomarker for GC patients.

It is noteworthy that there are 3 unavoidable flaws in the present report. Firstly, merely the TCGA database had PFS data, and the relationship of MATN3 expressing and PFS could not be confirmed in other databases. For that reason, it is difficult to complete a meta-analysis of PFS. Second, the expression of MATN3 was not confirmed in tumor specimens from our cohort, and in vitro and in vivo analyses were not conducted to explore the tumor-related function of MATN3 in GC progression. Eventually, despite the fact that our team finished the preliminary research pertaining to the biology processes of MATN3 in GC via enrichment assay, the mechanisms with more details linking MATN3 expressions with GC development need more assays biomedically.

## 5. Conclusion

Our findings unveiled that MATN3 expression was distinctly elevated in GC, and high MATN3 levels were related to cancer development and inferior prognostic results. Those discoveries showed that MATN3 might be a carcinogene in GC onset and progression and could be not only a new biomarker for prognosis but also an underlying treatment target for GC.

## Data Availability

The analyzed datasets generated during the study are available from the corresponding authors on reasonable request.

## Abbreviations

GC: Gastric cancer
MATN3: Matrilin-3
ECM: Extracellular matrix
TNM: Tumor node metastasis
ROC: Receiver operating characteristic
GEO: Gene Expression Omnibus
GO: Gene Ontology
MF: Molecular function
BP: Biological pathways
CC: Cellular components
AUC: Areas under the ROC curves
KEGG: Kyoto Encyclopedia of Genes and Genomes
OS: Overall survival
HR: Hazard ratio
CI: Confidence interval.
